# 

**Published:** 1831-04-01

**Authors:** 


### BIBLIOGRAPHICAL RECORD;

*Or Works Received for Review since last Quarter.*

1. A Manual of Surgery, founded upon the principles and practice lately taught by Sir Astley Cooper, Bart. F.R.S. Sergeant-Surgeon to the King, &c. and Joseph Green, Esq. F.R.S. &c. The Third Edition, considerably enlarged, containing many additional notes from the writings of other distinguished Surgeons. By Thomas Castle, F.L.S. of Queen's College, Oxford, Professor of Materia Medica to the Eclectic Society, &c. One Vol. 12mo. pp. 515. Price 10s. 6d.

☞ *This Edition forms a most valuable vade mecum of Surgery for the student and practitioner.*

---

2. A Catechism of Phrenology, illustrative of the Principles of that Science. By a Member of the Phrenological Society of Ed. Duodecimo, pp. 72. 1831.

---

3. A Treatise on Pathological Anatomy. By G. Andral, Professor, &c. translated from the French, by Richard Townsend, A.B. M.D. &c. and William West, A.M. M.D. &c. Volume the Second, (concluding volume) pp. 808. 1831.

☞ *We shall notice various subjects of this important Work, in our Review and Periscope.*

---

4. A Table of Vegetable Poisons, exhibiting the principal Poisonous Plants, comprising their common English name, Botanic name, the Class, Order, &c.—poisonous effects and mode of treatment, &c. illustrated by accurate drawings of the principal indigenous plants, including the poisonous mushrooms, &c. By G. Spratt, Surgeon, &c.

☞ *We had mislaid this very useful table, and beautiful engravings of the vegetable poisons, and hasten to repair the accident by strongly recommending it to the notice of our brethren.*

---

5. Illustrations of Mr. J. Cooper's Surgical Dictionary, published Monthly, &c. Parts, V. VI. &c.

☞ *This highly useful undertaking maintains its character, and should be in the possession of every one who has (and who has not ?) Mr. Cooper's Work.*

---

6. Article Surgery. Written for Brewster's Cyclopœdia. By JOHN LIZARS. In 1828. Quarto, bds. pp. 75—3 Plates.

☞ *This is a surprisingly good compendium of Surgery, considering the narrow limits to which the able author was confined.*

---

7. A System of Operative Surgery, containing a description of the most approved plans of performing the different operations in Surgery on the Dead Body; with Remarks on their Anatomy, Practical Observations: being principally designed for the use of Students in Surgery. By W. HARGRAVE, A.M. M.B. T.C.D. Lecturer on Anatomy, and Operative Surgery, &c. 12mo. pp. 533. Dublin, Hodges and Smith, 1831.

☞ *We may notice this useful Work in the body of the Journal.*

---

8. The Physiology of the Fœtus, Liver, and Spleen. By GEORGE CALVERT HOLLAND, M.D. &c. 8vo. pp. 229, London, 1831.

---

9. The Companion to Post-Mortem Examinations. Illustrated by Six Plates. Small Octavo, pp. 24. Rose, London, 1831.

---

10. Medical Zoology; and Mineralogy; or Illustrations and Descriptions of the Animals and Minerals employed in Medicine, and of the Preparations derived from them; comprising their generic and specific characters; English, Provincial, and foreign appellations: a copious list of Synomimes; Natural History; Physical, Chemical, and Medical Properties and Uses; including also a popular and scientific Account of Animal, Mineral, Atmospheric and Gaseous Poisons; with figures coloured from Nature, &c. By JOHN STEPHENSON, M.D. F.L.S. No. 1. To be continued monthly, price 3s. 6d. John Wilson, London, January, 1831.

---

11. First Principles of Medicine. By ARCHIBALD BILLING, M.D. Fellow of the Royal College of Physicians, &c. 8vo. pp. 131. 1831.

12. A Statistical Report of the Principal Provincial Hospitals in England, computed with reference to the latest Annual Returns of the Respective Institutions. 1830. By the REV. CHARLES OXENDON, Bishopsbourn, near Canterbury. 5 Sheets, Folio.

☞ *We admire Mr. Oxendon's zeal and industry. He has already incurred fifty pounds expence for gratuitous circulation of his statistical tables; but we advise him to be cautious in calculating on public patronage. These tables would, unquestionably, be very useful; but we doubt whether they will be much attended to by those who are addressed.*

---

13. The Effects of the Principal Arts, Trades, and Professions, and of Civic States and Habits of Living, on Health and Longevity: with a particular reference to the Trades and Manufactures of Leeds: 1831. By C. TURNER THACKRAH. Octavo, pp. 126.

---

14. Illustrations of Surgical Anatomy, with explanatory references, founded on the Work of M. Blandin. By JOHN G. M. BURT, Surgeon to the City Dispensary, &c. Edinburgh. No. I. and II.

☞ *This first fasciculus contains two plates of the Surgical Anatomy of the Neck, admirably engraved by the Messrs. Johnstones, of Edinburgh. The second fasciculus contains also two plates, one a Perpendicular Section of the Head and Neck, to shew the relative situations of the Cavities of the Nose, Mouth, Larynx, &c. The other plate delineates beautifully the different parts of the Eye. Each fasciculus is at the moderate expence of 2s. 6d.*

---

15. Manual of Operative Surgery, translated from the Third Edition of the French of J. Coster, M.D. By GEORGE FIFE, M.D. Surgeon to the Northern Public Dispensary, &c. Small 8vo. pp. 408. Edinburgh, 1831.

*** *M. Coster's Work is in considerable repute on the Continent. It exhibits in concise language the observations of all the most celebrated French Surgeons, as also the operations of Lisfranc, Dupuytren, Larrey, Roux, Lallemand, Maunoir, Vacca, Civiale, Heurteloup, Le Roy, &c. &c.*

---

16. Dissertatio Medica Inauguralis de Erysipelate. Auctore JOHANNE COLVAN, Armagh, 1829.

☞ *A learned and ingenious tentamen.*

17. The Report of the Board of Health in Manchester, June, 1830.

18. An Introductory Lecture, Delivered at the Hull General Infirmary, on Friday Nov. 12th, 1830. By JAMES ALDERSON, M.D. Fellow of the Royal College of Physicians of London, &c. 8vo. pp. 31, with Cuts. 1831.

19. An Introduction to the Study of Human Anatomy. By JAMES PAXTON, Member of the Royal College of Surgeons, and Author of the Illustrations of Paley's Natural Theology. 1 Vol. 8vo. pp. 415, with numerous Wood-cuts. Oxford and London, 1831.

☞ *This is a very interesting publication, and well calculated for the extra-professional but scientific reader.*

20. The Nottingham Dispensary, its Necessity, Origin, and Objects. By THOMAS JOWETT, Surgeon.

21. Professional Morality in 1831; or the Lawyer's Defence of Medical Quackery; in which St. John Long's Discoveries are Examined, and his Claims to the confidence of British Pulic are Criticized. By a Graduate of the University of Edinburgh. Second Edition. 8vo. sewed, pp. 86. Wilson, Princes-street, 1831.

22. The Pathology of the Fœtus, Liver, and Spleen. By GEORGE CALVERT HOLLAND, M.D. 8vo. London, 1831.

23. A Treatise on the Practice of Medicine, in Two Volumes. By JOHN EBERLE, M.D. &c. Philadelphia, 1830.

☞ *This is a very respectable compilation, infinitely superior to Thomas's Practice of Physic in this country.*

24. Observations on Fumigating, Vapour, and other Baths. By JONATHAN GREEN, Member of the Royal College of Surgeons.

⁂ *From personal observation, we can testify that Mr. Green's establishment is by far the most complete in London; and as it is carefully superintended by a regular surgeon, it deserves the patronage of the profession and the confidence of the public.*

25. An Address to the Medical Profession upon the Neglect of the Studies of Physiology and Morbid Anatomy. By R. WADE, M.R.C.S. and Lecturer on Morbid Anatomy, &c. 8vo. Sewed, pp. 16. March, 1831.

⁂ *A strong protest against indifference to the study of pathology.*

26. The Life of John Walker, M.D. late Director of the Royal Jennerian and London Vaccine Institutions. By JOHN EPPS, M.D. Lecturer on Materia Medica and Chemistry—Director of the Royal Jennerian and London Vaccine Institutions, &c. 8vo. pp. 342. March, 1831.

27. Address of Earl Stanhope, President of the Medico-Botanical Society, for the Anniversary Meeting, January, 1831. 8vo. Sewed.

⁂ *We wish we had a few hundred Earl Stanhopes among our Aristocracy!*

28. Cases of Lithotrity, or Examples of the Stone cured without Incision, &c. By BARON HEURTELOUP, M.D. 8vo. pp. 54. Sewed.

29. On Indigestion and Costiveness, with Hints to both Sexes, &c. By EDWARD JUKES, Surgeon. Small 8vo. pp. 160. March, 1831.

30. The Medical Annual for 1831. By R. REECE, M.D. 8vo. pp, 124. 1831.

⁂ *This is a popular work, and contains a great variety of intelligence which must render it very popular beyond the limits of the professien.*

31. Change of Air, or the Pursuit of Health; an Autumnal Excursion through France, Switzerland, and Italy, in the Year 1829; with Observations and Reflections on the Moral, Physical, and Medicinal Influence of Travelling-Exercise, Change of Scene, Foreign Skies, and Voluntary Expatriation, in Sickness and in Health. By JAMES JOHNSON, M.D. Physician Extraordinary to the KING. Octavo, pp. 300, March, 1831. Price 8s. 6d. bds. Highley and Underwood.

### INDEX.

A.

Abdominal aorta, aneurism of the.. 174
Abdominal pressure in obstetrics .. 244
Abercrombie, Dr. on the intellectual powers ................ 289
Abscess, fatal, from stricture....... 160
Abscess, encysted, of the spinal cord, 284
Abscess, fatal, of thigh ........... 516
Abuse of abstemious diet........... 154
Acrodynia, or Parisian epidemic ... 200
Acute rheumatism—pericarditis ... 166
Air, change of, in pursuit of health, 448
Albuminous urine, Dr. Elliotson on, 513
Alderson, Dr. on hooping-cough ... 142
Anatomical engravings, Dr. Knox's, 245
Anatomical plates, M. Schloss' .... 566
Anatomy, Bransby Cooper's lectures 140
Anatomy of the testis, Sir A. Cooper 375
Andral's pathological anatomy .... 434
Aneurism of the external iliac—aorta tied.......................... 50
Aneurism, common iliac tied...... 54
Aneurism, diffused, case of........ 488
Anterior cerebral lobes, congenital abscess of ...................... 195
Anus and rectum, Dr. Colles on 218
Aorta, compression of, in uterine hæmorrhage .................... 152
Aorta, rupture of semilunar valves 197
Apennines, scenery of............. 454
Apoplexy, from ruptured middle cerebral artery ................ 234
Apoplexy of the lungs, two cases of, 360
Apothecaries' Company, answers to queries ...................... 256
Arbitrary association of ideas...... 300
Armstrong, Dr. the late .......... 242
Arterial system, diseases of the.... 173
Arts, trades, &c. their influence .. 312
Asthma, Grinders', Dr. Knight on 202
Atmospheric air, in deafness ...... 469
Auscultation, evidence of pregnancy 465
Auscultation in pregnancy........ 495

B.

Barry, Dr. on the Gibraltar epidemic 69
Barry, Dr. his letter to Sir J. M'Grigor ..................... 246
Billing, Dr. his principles of medicine .......................... 391
Bleeding, diffused aneurism from .. 282
Blood, concrete oil in the ........ 208
Boivin, Mad. on supposed rheumatism ........................ 261
Bracco, a magnificent mountain-pass 461
Brachet, M. on the ganglionic system 33
Brain, abscess of the ............. 233
Brain, inflamed, from old injury of head ........................ 231
Brain, tumours in................. 545
Brodie, Mr. his recto-vesical lithotomy .......................... 187
Buchanan, Dr. on lithoplatomy.... 265

C.

Cancer, Sir E. Home on ........... 44
Cancer, sarsaparilla for .......... 48
Cancer of the jaws, Chirurgus on.. 210
Cancer uteri, Dr. Montgomery on.. 266
Captain Moir, trial of ............ 150
Care-worn countenance .......... 449
Carpo-pedal spasm, case of........ 366
Castration, operation of .......... 11
Catarrhal ophthalmia ............ 401
Cerebellum, abscess of............ 547
Cerebral artery, middle, ruptured.. 234
Change of air, Dr. Johnson on .... 448
Chest, rheumatic affection of the .. 489
Chimney-sweeper's cancer, Sir A. Cooper on ..................... 29
Chlorine in consumption ........ 103
Cholera morbus in Russia ........ 191
Cholera of India, Dr. Smith on.... 347
Cholera, nature of ............... 348
Cholera, symptoms of............. 350
Cholera, treatment of............ 350
Chymification, derangements of ... 438
City of London Medico-Chirurgical Society ....................... 192
Climate of Malta ................. 336
Clinical Review, preface to the.... 155
Cold, its action on the lungs ...... 147
Cold-taking, remarks on ........ 312
Coliseum, reflections on.......... 458
College of Surgeons of Dublin .... 239
Colles, Dr. on diseases of the anus 218
Collins, Dr. on laceration of uterus 357
Colon, cancer of its lower portion.. 172
Combe, Mr. on phrenology........ 321
Concussion of the brain, case of .. 278
Contagion of plague.............. 190
Contagious ophthalmia .......... 403
Convulsion, peculiar, Dr. M. Hall on 366
Convulsive croup, article on ...... 366
Cooper, Sir Astley, on the testis.. 1
Cooper's, Bransby, lectures ...... 140
Cooper, Sir Astley, on the testis.... 375
Corfu, climate, &c. of............ 97
Corfu, diseases in................ 101
Corneitis, scrofulous ............ 421
Crampton, Mr. his ligature of the common iliac.................. 54
Crampton, Dr. on melanosis ...... 363
Cranium, caries of ............. 546

Cretinism, Dr. Johnson on........ 452
Cutaneous diseases, severe, on .... 477
Cynanche, fatal case of .......... 171

D.

Darwall, Dr. on a peculiar paralysis, 249
Davy, Sir Humphry, life of .... . 423
Death from loss of blood.......... 491
Democritus, jun. on quackery .... 193
Depôt in one arm, from scalp-wound 236
Depôts in distant parts, from erysipelas ...................... 237
Diet, abstemious, abuse of........ 154
Diffused spurious aneurism, case of, 282
Diffused aneurism, case of........ 488
Diseases of the eye, Mr. Mackenzie 397
Dropsy, Dr. Billing's views of .... 395
Dropsy, Dr. Elliotson on.......... 511
Dublin College of Surgeons ....... 239
Dublin Medical Transactions ..... 357
Dysentery, large doses of ipecacuan in ......................... 332
Dyspnœa, spasmodic, cured by purging ......................... 163

E.

Ear, internal, disease of the ...... 232
Elephant, fury fits of the ........ 254
Elliotson, Dr. on diseases of the heart ......................... 60
Elliotson, Dr. on glanders in man.. 108
Elliotson, Dr. on paralysis agitans, 167
Elliotson, Dr. on diseases of the heart ......................... 323
Elliotson, Dr. on dropsy .......... 511
Empyema, case of .............. 520
Engravings of the bones, arteries, and nerves.................... 245
Epidemic at Gibraltar, Dr. Barry on 69
Epididymis, anatomy of the ...... 382
Epistaxis, fatal, case of .......... 165
Ergot of Rye, use of ............ 479
Erysipelas of the scalp, cases of.... 180
Erysipelas, followed by depôts, &c. 237
Erysipelas of the scalp, case of ... 274
Erysipelatous ophthalmia ........ 413
Etiolation, human .............. 450
Eustachian tube, chronic inflammation of ...................... 469
Eye, Mr. Lawrence on the venereal diseases of the ................ 80
Eye, Mr. Lawrence on venereal diseases of the .................. 116
Eye, Mr. Mackenzie on the diseases of the......................... 397
Eyelids, syphilitic ulceration of the, 135

F.

Facts, study of .................. 308
Facts, generalization of .......... 310
Fever at Gibraltar, Dr. Gilkrest on 224
Fever, state of intestinal canal in .. 443
Fever, rapidly fatal case of ........ 472
Fingers and toes, Dr. Rynd on the, 214
Fistula ani—a *fistulous* report ..... 157
Fistula perinei, report on ........ 501
Florence, climate of.............. 455
Fractured thigh, fatal case of .... 228
Fractured leg, non-union of ...... 229
France, characteristics of ........ 450
French operations................ 564
Fungoid disease of the testis ...... 1
Fungoid disease of the testis ...... 263

G.

Ganglionic system, researches on the 33
Ganglionic system, general and special functions of .............. 34
Ganglionic system, its influence on various organs ................ 35
Gangrene of the leg from scalp-wound ........................ 235
Gangrenous erysipelas............ 499
Geneva, characteristics of ........ 452
Genoa to Nice, new road.......... 462
Gibraltar epidemic, Dr. Barry on the, 69
Gibraltar fever, Dr. Gilkrest on the, 224
Gibraltar fever; code sanitaire .... 246
Glanders in the human subject .... 108
Glottis, spasm of, Dr. Marsh on.... 366
Goitre.......................... 453
Gonorrhœal inflammation of the eye 82
Gonorrhœal ophthalmia, treatment 88
Gonorrhœal ophthalmia, cases of .. 91
Grinder's asthma, Dr. Knight on the 202
Guaco, Mr. Hawkins on .......... 571

H.

Hæmatocele, Sir A. Cooper on ..... 26
Hanging, effects of the rope in .... 345
Harrison, Dr. on sterility ........ 470
Head, report on injuries of the .... 274
Heart, Dr. Elliotson on the diseases 60
Heart, affections of its lining membrane ........................ 60
Heart, on the sounds of the ...... 61
Heart, disease of the auriculo-ventricular openings of............ 64
Heart, remarkable disease of the .. 196
Heart, malformation of the ...... 231
Heart, Dr. Elliotson on diseases of the 323
Heart, sounds of, in disease........ 325
Heart, diseased, general symptoms of 327
Heart, rupture of the ............ 330
Heart, neuralgia of the .......... 330
Heart, anomalous state of the ..... 364
Heart, disease of the .. ......... 521
Hematemesis, its causes in the liver 548
Hemiplegia, treatment of ........ 477
Hemiplegia, intermittent, case of .. 514
Hemiplegia, with and without visible cause ...................... 547

Hennen, Dr. his medical topography 97
Hennen, Dr. on the climate of Malta 336
Hepatic dysentery .............. 334
Hernia, Mr. Geoghegan on......... 149
Home, Sir E. on Tumours ........ 41
Hooping-cough, pathology of...... 142
Houston, Dr. on the rectal mucous membrane .................... 213
Houston, Dr. his pathological observations ...................... 344
Human subject, glanders in the.... 108
Hunger and thirst; state of alimentary canal ..................... 437
Hunt, Mr. his operation for ptosis.. 243
Huskisson, Mr. on his death ...... 198
Hydatid disease of testis .......... 200
Hydrocele, Sir A. Cooper on ...... 14
Hydrocele, modes of treatment for, 18
Hydrocele, of the spermatic cord .. 22
Hydro-rachitis, case of ........... 162
Hydrocephalus, encysted.......... 346
Hypertrophy of the heart.......... 323

I.

Ileus, cases of .................. 509
Iliac aneurism—aorta tied ........ 50
Iliac artery (common) tied by Mr. Crampton ..................... 54
Incontinence of urine, cure of .... 569
Indian cholera, Dr. Smith on...... 347
Inflammation of the tunica vaginalis 23
Inflammation, theory of.......... 393
Inflammation of brain, from old injury.......................... 231
Inhalation of chlorine in consumption .......................... 103
Inhalation in phthisis, Sir C. Scudamore on ..................... 352
Injury of the spine, cases of ..... 226
Injuries of the head, hospital report on ........................... 274
Insanity, theory of .............. 304
Intellectual powers, Dr. Abercrombie on the ..................... 289
Intermittent gastro-enteralgia .... 468
Intermittent hemiplegia.......... 514
Internal iliac; dissection of Dr. Stevens' case.................. 57
Inverted uterus, extirpation of .... 251
Ionian Islands, medical topography of the........................ 97
Ipecacuan, large doses of in dysentery.......................... 332
Iritis, syphilitic, Mr. Lawrence on, 116
Iritis, syphilitic, symptoms of ..... 116
Iritis, syphilitic, effects of ........ 120
Iritis, syphilitic, in infants........ 122
Iritis, syphilitic, treatment of...... 123
Iritis, syphilitic, turpentine in .... 127
Iritis, syphilitic, cases of.......... 128
Ischuria renalis, case of .......... 165
Italy, climate of ................ 148
Italy, characteristics of .......... 459
Italy, climate of ................ 462

J.

James, Mr. his ligature of the aorta, 50
John St. John Long.............. 241
Johnson, Dr. on change of air .... 448

L.

Laceration of uterus and vagina .. 357
Lambert, the late Mr............ 478
Lancetted stilettes in stricture .... 195
Laryngitis, chronic; tracheotomy successful ..................... 176
Larynx, œdema of, tracheotomy ... 500
Lawrence, Mr. on the venereal diseases of the eye................ 80
Lawrence, Mr. on the same ....... 116
Lawrence, Mr. on inflammation of veins ........................ 212
Life of Sir Humphry Davy ........ 423
Liniment of St. John Long........ 257
Lithoplatomy, Dr. Buchanan on .. 265
Lithotomy, recto-vesical, case of .. 187
Lithotrity—Mr. Costello.......... 569
Liver, state of, in icterus.......... 494
London College of Medicine ...... 572
Loss of blood, death from ........ 491
Lumbricus, escape of, through perforated stomach ............... 364
Lungs, action of cold on the ...... 147
Lungs, putrefactive disorganization 549

M.

Mackenzie, Mr. on diseases of the eye ......................... 397
M'Kinnal, Dr. on a malignant yellow fever ..................... 38
Malaria of Italy ................ 457
Malignancy of tumours at first innocent ....................... 521
Malta, Dr. Hennen on the climate of 336
Malta, endemic diseases of........ 338
Malta, plague at ................ 339
Malta, pulmonary affections at.... 342
Materialists, arguments against the, 292
Medical topography of Ionian Islands 97
Medical seed-time .............. 145
Medical botany.................. 286
Medical practitioners, longevity of, 319
Medical reform.................. 573
Medicine, Dr. Billing's principles of, 391
Medico-Chirurgical Transactions .. 49
Melanosis, case of .............. 363
Mental illusions ................ 294
Merchants and manufacturers, longevity of ..................... 318
Mind, progress of disease on the ... 301
Miracles, arguments against ...... 298
Mitral valves, ossification of ...... 567

Montgomery, Dr. on cancer uteri.. 266
Morality among the ancient Romans 460
Moxa, expeditious................ 258
Mucous membrane of the rectum, 213
Murder and suicide, propensity to.. 254

N.

Nævus, new mode of treating ..... 480
Nails, diseases of the ............ 216
Naval surgeons—court etiquette ... 570
Neuralgia, and tumours of nerves.. 161
Norwich Medical School.......... 248
Nothing new under the sun ...... 467
Nuttall, Dr. the late ............. 286

O.

Obstruction of the cava and iliac veins ........................ 206
Occipito-frontalis ; diffuse inflammation ........................ 183
Œsophagus, foreign bodies in...... 565
Oil in the blood, Dr. Babington on, 208
Ophthalmia, gonorrhœal, acute.... 82
Ophthalmia, gonorrhœal, cases of.. 91
Ophthalmia, catarrhal............. 401
Ophthalmia, contagious ........... 403
Ophthalmia, neonatorum ........ 407
Ophthalmia, scrofulous .......... 409
Ophthalmia, erysipelatous ........ 413
Ophthalmia, variolous............ 414
Ophthalmia, morbillous and scarlatinous......................... 416
Ophthalmia, rheumatic .......... 417
Ophthalmia, catarrho-rheumatic .. 419
Ophthalmiæ in general .......... 397
Ophthalmiæ, treatment of the .... 399
Ovarian disease, remarkable case of 552

P.

Painful swellings of the lower extremities......................... 496
Paralysis, Dr. Darwall on a species of 249
Paralysis agitans, remarks on, and case of ...................... 167
Paralysis agitans, Mr. Parkinson's cases of ...................... 170
Paris, Dr. his life of Davy ........ 423
Parisian epidemic, description of the 200
Pathological observations, Dr. Houston's ...................... 344
Pathological anatomy, Andral's.... 434
Pathology of hooping cough ...... 142
Pellagra of Lombardy............ 453
Pericarditis from acute rheumatism 166
Perineal fistula, report on ........ 501
Perineal fistula, remarks on ...... 505
Periostitis, Dr. Weir's report on.... 481
Phlegmatia dolens — inflamed veins 212
Phrenology, Mr. Combe on........ 321
Phthisis, inhalation of chlorine in.. 103
Plague at Malta ................ 339
Plethora from suppressed evacuation 487
Pneumonia biliosa, Mr. Allen on .. 262
Pneumo-thorax, Mr. Spittal on .... 507
Pompeii, reflections on .......... 460
Pontine fens, passage of .......... 459
Pregnancy, auscultation evidence of 465
Pregnancy, auscultation in........ 495
Professional omnibuses .......... 145
Professional Morality in 1831 .... 467
Prolapsus uteri, Baron Larrey on .. 259
Psoriasis, treated by depletion .... 285
Ptosis, new operation for.......... 243
Pulmonary affections at Malta .... 342
Pulmonary apoplexy, two cases of 360

Q.

Quackery, Democritus, junr. on .. 193
Quain, Mr. his introductory lectures 260

R.

Recto-vesical lithotomy, Mr. Brodie's 187
Rectum, organic stricture of the .. 218
Rectum, cancer of the............ 220
Rectum, vascular tumours of the .. 222
Rectum, ulcer of the ............ 223
Reflection, ideas derived from .... 296
Reform, medical ................ 573
Rete mucosum, description of the.. 141
Retention of urine, cases of ...... 159
Retention of urine, fatal .......... 471
Rheumatic ophthalmia .......... 417
Rheumatic affection of the chest .. 489
Ruspini's styptic, composition of .. 480
Russian rubles, for medical essayists 192
Rynd, Dr. on affections of the fingers and toes.................. 214

S.

St. John Long again upon the scene 241
St. John Long, his liniment ...... 257
Scalp wounds and their consequences 178
Scalp wounds, cases of............ 179
Scalp wounds, followed by erysipelas 180
Scalp, diffuse inflammation of the.. 183
Scalp wounds, and their consequences 235
Scalp wound, case of.............. 280
Sciatica, cured by acupuncture .... 477
Scirrhous disease of the testis...... 9
Scrofulous ophthalmia............ 409
Scrotum, cancer of the .......... 29
Scudamore, Sir C. on phthisis...... 352
Semilunar aortic valves ruptured, 197
Senses, fallacious evidence of the .. 298
Simplon, night on the ............ 453
Singleton's golden ointment ...... 285
Spasm of the glottis, Dr Marsh on.. 366
Spasmodic dyspnœa cured by purging 163
Spasmodic cholera, Dr. Smith on .. 347
Spermatic cord, anatomy of the .. 383
Spine, cases of injury of the ...... 226
Spine, disease of the.............. 518

Spine, fractured, paralysis from.... 519
Stammering cured by purgatives .. 209
Stephens, Dr. his ligature of the internal iliac .................. 57
Sterility, Dr. Harrison on ........ 470
Stomach, case of perforation of the 364
Strictures, lancetted stilettes in.... 195
Suppressed evacuations, plethora from ........................ 487
Suppression of urine for seven weeks 468
Surgical and pharmaceutical practice.......................... 570
Sybille, malignant yellow fever in the .......................... 38
Syphilitic iritis, Mr. Lawrence on.. 116
Syphilitic ulceration of the eyelids 135

T.

Testis, Sir A. Cooper on the ...... 1
Testis, fungoid disease of the...... 1
Testis, scirrhous disease of the .... 9
Testis, venereal inflammation of the 194
Testis, hydatid disease of the ...... 200
Testis, fungoid disease of the...... 263
Testis, Sir A. Cooper on the ...... 375
Testis, description of the ........ 378
Testis, descent of the ............ 386
Testis, on chronic inflammation of 554
Thackrah, on the effects of Trades, &c. on health ................ 312
Thomson, Dr. his reclamation .... 153
Throat, wounds of the............ 473
Tobacco, use of.................. 315
Tracheotomy successful for chronic laryngitis .................... 176
Trades, &c. their effects on health 312
Traumatic tetanus, report ........ 157
Traumatic tetanus, cured by turpentine.......................... 477
Trial of Captain Moir............ 150
Truth, Dr. Abercrombie on ...... 289
Tumours, Sir E. Home on ........ 41
Tumours becoming malignant; cases 521
Tunica vaginalis, inflammation of.. 23
Tunica vaginalis, cartilaginous bodies in the.................... 24
Tunica vaginalis, ossification of the 25
Tunica vaginalis, fungoid inflammation of the.................... 25
Twining, Mr. on dysentery........ 332

U.

Uterine hæmorrhage, compression of the aorta in .. .............. 152
Uterus, inverted, extirpation of ... 251
Uterus, Dr. Montgomery on cancer of the........................ 266
Uterus and vagina, laceration of .. 357
Uterus, case of cancer of the .... 475

V.

Varicocele, Sir A. Cooper on ...... 28
Variolous ophthalmia ............ 414
Veins, obstruction of the, in phthisis 206
Venereal diseases of the eye, Mr. Lawrence on.................. 80
Venereal diseases of the eye, Mr. Lawrence on.................. 116
Venereal inflammation of the testis 194
Venus de Medicis................ 456

W.

Wear and tear, Dr. Johnson on.... 449
Weir, Dr. on periostitis .......... 481
Wounds of the scalp and their consequences .................... 178
Wounds of the scalp and their consequences .................... 235
Wounds of the throat, M. Larrey on 473
Wright, Dr. and Bethlem Hospital 471

Y.

Yellow fever, malignant, in the Sybille ........................ 38

---

### TO CORRESPONDENTS.

We do not think that the cheap translations of Louis, Andral, Bertin, and some other French authors, suggested by P——h, would pay the expense of publication.

We have not heard any thing of the projected general meeting of the French Medical Corps. General politics are now engrossing them more than medical reform.

The review of Dr. Coombe's work on mental derangement was unavoidably postponed till next number, when it will certainly appear.

The additional observation (10th) on Spasmodic Cholera, came to hand after the others were printed off. The Prophylaxis, however, offers little that is satisfactory in the present state of our knowledge.

Mr. Lizar's observations on Hernia were published in another journal about the time we received them from the author.

The communication of S. respecting the late Dr. Armstrong has come to hand, and we have little doubt but that *his* statement is the more correct version of the election to the Fever Hospital. "There were two candidates, but the feeling of the committee was in favour of Dr. Armstrong. The election was arranged to suit the expected admission of Dr. A. as a licentiate. The committee met, and waited a considerable time for Dr. A. who, at length, came into the room, and announced that he must withdraw, as he had just been rejected at the College. The whole Committee were surprised and astonished. Considering it hard, however, that Dr. A. should be thus disappointed, and unwilling to accept the other candidate, it was moved and carried that the bye-law, confining the office to fellows and licentiates, should be suspended. By these means the election of Dr. A. was effected."—S.

Dr. Holbrook, of Cheltenham, informs us that the following formula corresponds in sensible properties with a specimen of Long's liniment. R. Plumbi superacet. 1 dr. Acid. acet. fort., Spir. terebinth. aa 1 oz. Misce fiat linimentum. We have no doubt that the Charlatan of Harley Street has several kinds of liniment, though he pretends that one only is necessary for the cure of all diseases.

Mr. Ceeley, of Applesbury, in a letter dated 13th February, 1831, observes as follows, respecting the nitrate of silver. "I have had lately astonishing results from the internal use of nitrate of silver, in several cases of gastric and intestinal derangements of long standing. I am satisfied that the efficacy of this article in such cases, is not sufficiently known to the profession. Scarcely a day passes that I do not prescribe it internally, with the happiest effects."

---

### DR. JOHNSON ON CHANGE OF AIR.

CHANGE of AIR, or the PURSUIT of HEALTH; an Autumnal Excursion through France, Switzerland, and Italy, in the Year 1829; with Observations and Reflections on the Moral, Physical, and Medicinal Influence of Travelling-Exercise, Change of Scene, Foreign Skies, and Voluntary Expatriation, in Sickness and in Health. By JAMES JOHNSON, M.D. Physician Extraordinary to the KING. Octavo, pp. 300, March, 1831. Price 8s. 6d. bds. Highley and Underwood.

### CRITICISMS ON THE ABOVE WORK.

"To attempt an analysis of a work embracing such a treasure of anecdote and instruction would be worse than an idle task; we must therefore content ourselves with observing that, of all the popular tours, of which British literature has been recently so prolific, this is immeasurably the best, whether we consider it in point of style or details. There is no class of general readers which may not derive pleasure and profit from its perusal; while to the physician it will prove very useful, whether as to his guidance in the selection of suitable residences, or routes for patients seeking the renovation of health—or as to the history of those diseases which the malarious atmosphere of Italy, or the Alpine blasts of Switzerland too frequently induce."—*Ballot*, *27th Feb.* 1831.

"Dr. Johnson's book contains much curious information on the subject of CHANGE of AIR, and on the causes of ill health in cities. To the justice of his observations experience will enable every reader of mature judgment to subscribe. His recommendations appear to be very judicious."—*Sunday Times*, *27th Feb.* 1831.

"The author spent a few months in skimming lightly over France and Switzerland, dwelling with industrious detail on Italy, and its myriad sources of curiosity and interest. The Doctor does not pretend to paint externals. He travels *en philosophe*, and has attended more to the *morale* than the *physique* of what he sees. He is, however, a vigorous independent thinker, while his opinions are slightly tinctured with cynicism, which gives them an agreeable relish. His style is clear, bold, and expressive; so that when he least appears to aim at effect, he leaves a more vivid impression on us of the object of his reflections, than others would by an elaborate description. His ponderings over the ruins of empires, notwithstanding the lucubrations of all who have pondered on the same subject, are generally both instructive and amusing—never tiresome. But there is no part of his book which possesses more interest, though of a painful kind, than where he describes the deadly effects of that perpetual scourge, not only of the most barren, but of the most lovely and fertile portions of the garden of the world—MALARIA. The Doctor deserves credit also for the forbearance of his single volume."—*Morning Herald*, 16th March.

"The author, out of his abundant stock of reflection and knowledge, has constructed a volume with which all classes will be pleased—some for its amusing qualities—others for its really useful information—and a still larger class for its combination of both."—*Atlas*, *March 20th*, 1831.

